# Zika discourse in the Americas: A multilingual topic analysis of Twitter

**DOI:** 10.1371/journal.pone.0216922

**Published:** 2019-05-23

**Authors:** Dasha Pruss, Yoshinari Fujinuma, Ashlynn R. Daughton, Michael J. Paul, Brad Arnot, Danielle Albers Szafir, Jordan Boyd-Graber

**Affiliations:** 1 Department of History and Philosophy of Science, University of Pittsburgh, Pittsburgh, PA, United States of America; 2 Department of Computer Science, University of Colorado, Boulder, CO, United States of America; 3 Department of Information Science, University of Colorado, Boulder, CO, United States of America; 4 Analytics, Intelligence, and Technology Division, Los Alamos National Laboratory, Los Alamos, NM, United States of America; 5 Department of Computer Science, University of Maryland, College Park, MD, United States of America; Dalian University of Technology, CHINA

## Abstract

This work examines Twitter discussion surrounding the 2015 outbreak of Zika, a virus that is most often mild but has been associated with serious birth defects and neurological syndromes. We introduce and analyze a collection of 3.9 million tweets mentioning Zika geolocated to North and South America, where the virus is most prevalent. Using a multilingual topic model, we automatically identify and extract the key topics of discussion across the dataset in English, Spanish, and Portuguese. We examine the variation in Twitter activity across time and location, finding that rises in activity tend to follow to major events, and geographic rates of Zika-related discussion are moderately correlated with Zika incidence (*ρ* = .398).

## Introduction

In early 2015, a large outbreak of the Zika virus started in Brazil. Soon, areas affected by the virus began experiencing higher-than-normal rates of microcephaly, a birth defect in which infants are born with abnormally small heads, and Guillain-Barré syndrome, a nervous system disorder in which muscle weakness and paralysis results from nerve cells being attacked by the immune system. In November 2015, Brazil declared a public health emergency; by February 2016, the mosquito-borne virus had spread to more than 20 other countries and territories in the Americas and was declared a Public Health Emergency of International Concern by the World Health Organization (WHO) [1]. This outbreak has resulted in widespread public concern and international media coverage [2].

During an emerging outbreak like this, it is important to communicate to the public information about the spread of the disease along with advisories on how to protect against the disease. At the same time, it is important to understand how the public responds to and receives information about the outbreak because this sheds light on how an epidemic spreads, how information can be disseminated to the public, and how the public views proposed interventions. Understanding how the public responds to and receives information about the outbreak is important for helping understand how an epidemic spreads [3, 4], how information can be disseminated to the public [5], and how the public views proposed interventions [6].

Much of this information, including the thoughts and reactions of individuals, is communicated over social media platforms like Twitter. While not without limitations as a source of data, social media can provide a rich perspective into the information landscape surrounding Zika. In this study, we analyze Twitter to understand when, where, and what people share about Zika online. Our dataset contains every tweet mentioning Zika from March 2015 to October 2016, with our analysis focusing on a subset of nearly 4 million tweets geolocated to countries and territories in the Americas. We utilize a multilingual topic model [7] to automatically extract the major themes or topics of discussion aligned across three languages: English, Spanish, and Portuguese. We then examine the spatiotemporal patterns of these topics, including the rise and fall of topics in relation to events, and topic prevalence by country.

### Background

#### A brief history of the Zika virus and its 2015-16 outbreak

The Zika virus is a disease that typically causes minor flu-like symptoms. However, the virus is also associated with more serious medical conditions, including microcephaly, a birth defect in which infants are born with abnormally small heads, and Guillain-Barré syndrome, a nervous system disorder in which muscle weakness and paralysis results from nerve cells being attacked by the immune system [8]. The Zika virus was first identified in humans living in Uganda and the United Republic of Tanzania in 1952. The virus received little attention until it was linked with Guillain-Barré syndrome during an outbreak in French Polynesia in 2013-2014 [9].

In 2015, a Zika virus outbreak hit a region in northwest Brazil. Increasing numbers of microcephaly were soon reported in the regions affected by the virus, and in October, the Brazil Ministry of Health confirmed that the rates of microcephaly in the areas affected by the virus were higher than in the rest of Brazil [10]. Many of the women who gave birth to microcephalic infants had detectable levels of the Zika virus in their amniotic fluid, suggesting that they had been ill with Zika during their pregnancy. Guillain-Barré syndrome was also reported in higher numbers in Zika-affected regions. In February 2016, the World Health Organization (WHO) declared the Zika outbreak a Public Health Emergency of International Concern. No effective anti-viral medication or vaccine exists for combating the disease.

Zika is primarily transmitted by *Aedes* mosquitoes, a species native to parts of the central Americas, though in some cases it can also be transmitted sexually or from a pregnant woman to her fetus [2, 11]. Because mosquito bites are the most common cause of Zika transmission, the spread of the virus is more geographically constrained than diseases like flu because it is not transmitted by infected people in public spaces and is only prevalent in regions native to *Aedes*. Because of this, Zika’s effects have largely been confined to the Americas and have primarily spread through parts of South, Central, and North America, where this species of mosquito lives.

#### Social media and epidemiology

The increasing prevalence of digital data sources, like electronic health records, has helped overcome the challenge of running decades-long epidemiological studies [12]. One such source of data is social media, and in particular Twitter, which is being increasingly used as a supplementary data source in epidemiology and other areas of public health [13]. Comparing tweets about disease to gold-standard incidence data is a popular methodology in the computational epidemiology community. For example, several studies have found correlations between the volume of flu-symptom related tweets and official government statistics from sources like the Centers for Disease Control and Prevention (CDC) and the Health Protection Agency [14, 15], and similarly, tweets have been used to measure dengue fever—another mosquito-transmitted virus—in Brazil [16]. Combining social media data with traditional hospital-collected data can improve disease surveillance and forecasting beyond using either data source alone [17, 18]. These and other methods of analyzing social media data provide policymakers and health professionals with rich information about population health.

Beyond monitoring the prevalence of a disease, social media data can provide insights into the public’s awareness of, reactions to, and concerns about disease [19, 20]. For example, Mollema *et al*. [21] characterized reactions to the 2013 measles outbreak in the Netherlands, and a number of researchers studied reactions to the 2014 ebola outbreak in Africa, particularly focusing on anxieties in the US [22–24]. Our study is related to this type of work, as most tweets about Zika communicate information about the disease or reactions to this information, rather than personal experiences with the disease.

#### Zika and Twitter: Existing work

A small number of recent studies have examined Zika-related discussion in Twitter data. McGough *et al*. [25] found that tweets, along with Google Trends data, can improve forecasting of Zika incidence. Other studies have typically focused on one of two aims, both of which are captured by our study: understanding the content of tweets, and analyzing spatiotemporal patterns in tweets. Miller *et al*. [26] and Vijaykumar *et al*. [27] both studied the content of Twitter messages, identifying the major themes of discussion; however, both studies had the limitation of focusing on English-language tweets, which misses the majority of tweets from Latin America. Rather than focusing on content, Stefanidis *et al*. [28] and Bragazzi *et al*. [29] characterize patterns in social media activity over time and geography. Our work extends these studies by combining content analysis with spatiotemporal analysis and by including content across multiple languages (English, Spanish, and Portuguese).

#### Topic modeling for text analysis

Text mining techniques can automatically extract the major themes of a large text corpus, allowing researchers to conduct a content analysis at scale [30]. Various methods exist that identify themes based on statistical patterns in the text, such as the co-occurrences of different words [31]. *Topic models* are one method of co-occurrence analysis that cluster related words together into “topics” and assign topics to documents [32]. This approach allows one to characterize the topic content of a dataset, as well as identify patterns in the distribution of topics across documents.

Most topic models are based on Latent Dirichlet Allocation (LDA) [33], a probabilistic model that has been successfully used to measure health content in tweets [34]. In LDA, each document is modeled as a probability distribution over latent topics, while each topic is defined by a probability distribution over words. The parameters of LDA-based models, which are automatically inferred from the data, are regularized with Dirichlet priors.

A number of public health studies using social media data have used topic models in their analyses, including for identifying infectious disease tweets [35, 36], understanding healthcare reviews [37, 38], and studying health behaviors [39–41].

### Contributions and overview

Our contributions include a new dataset, a new topic modeling technique, and new insights into the spread and composition of Zika information in social media. Specifically:

- We introduce a dataset containing over 15 million tweets mentioning Zika spanning March 2015 through October 2016. We share the tweet IDs from this dataset, along with location, language, and topic information.
- We extend prior work in multilingual topic modeling for non-parallel corpora and show how it can be used to understand non-English infectious disease tweets. We also find that crosslingually coherent topics can be learned with only a partial set of translations between languages, indicating this is a scalable method that could be used in other public health contexts.
- We characterize the content of Zika-related tweets and show how content and volume vary across time and location. We find that Twitter discussion rises in response to significant news and events; tweets are most prevalent in areas most strongly affected by Zika; and areas most affected by Zika are more likely to discuss topics like research and morbidities of the disease, while areas less affected or unaffected by Zika are more likely to discuss peripheral topics like politics and the 2016 Olympics.

## Materials and methods

### Data

We describe an initial collection of over 15 million tweets, covering 188 countries in 97 languages. Our study focuses on a subset of 3.9 million Zika-related tweets geolocated to North and South America. Our topic analyses focus on a narrower subset of 3.7 million tweets that are in English, Spanish, or Portuguese.

#### Zika tweets

This study uses 14.3 million tweets mentioning “zika” or “ZIKV” (abbreviation of “Zika Virus”) from March 1, 2015 to October 31, 2016, collected in accordance with the Twitter terms of service. The data were collected from Gnip, a commercial service that sells tweets for a fee. Specifically, the tweets were requested using Gnip’s Historical PowerTrack API, which provides the full collection of tweets matching these criteria.

The data also includes an additional 1.2 million tweets that mention “zica,” an alternative spelling of Zika in Brazilian Portuguese. However, this word also has other meanings. We include these tweets when sharing our dataset, but we excluded these from this study to avoid ambiguity. Once removing “zica” tweets, we did not observe many irrelevant tweets in the dataset, though we note that this type of keyword filtering is limited because there is a possibility of excluding tweets that discuss Zika indirectly without an explicit mention [42].

#### Control tweets

We also use a collection of 42.1 million tweets randomly sampled from Twitter using the streaming API, spanning approximately 10 days throughout December 2017 and January 2018. From this sample, we proportionally estimate the overall number of tweets in each location, which we use to create per-capita estimates of tweet volume. The details of obtaining location information from these tweets are explained in the next subsection.

### Attribute inference

For each tweet, we extracted two types of metadata: location and language.

#### Location

We geolocate tweets using Carmen [43], a tool that uses a combination of geo-coordinates, Twitter Place attributes, and user profile information, depending on which is available, to infer a location for a tweet. At the country level, which is the level used in our study, the accuracy of Carmen is estimated to be 90%. Carmen was able to resolve 34.9% of tweets in our collection to a country. The majority of tweets are geolocated using the user profile, which means that the location most likely represents the “home” location of the user rather than the current location when the tweet was posted [43]. Thus, our geographic analysis is more likely to reflect the nationalities of the users than their locations when tweeting (e.g., vacationers visiting other countries).

Our study is restricted to the 3.9 million tweets that were geolocated to the Americas. Table 1 shows the locations included in our analysis. We treat territories as their own country if they have an ISO country code; for example, we treat the US territory of Puerto Rico as its own country. Some locations, such as French Guiana, are not recognized by Carmen and are not represented in the dataset.

**Table 1:** The number of tweets from each country or territory in our Americas dataset, along with the percentage in each language.

*Country/Territory*	*# Tweets*	*%* en	*%* es	*%* pt
United States	2,275,072	90.69	4.23	0.91
Venezuela	402,489	3.45	90.93	0.58
Brazil	392,600	9.92	3.24	68.67
Canada	121,412	91.26	1.72	0.59
Mexico	98,121	11.10	80.77	0.66
Argentina	94,508	14.07	78.20	1.08
Colombia	93,338	10.12	83.37	0.62
Chile	55,376	8.54	85.92	0.71
Dominican Republic	48,199	8.82	85.63	0.46
Ecuador	47,415	8.24	87.96	0.42
Puerto Rico	41,032	25.33	67.67	1.96
Honduras	34,747	4.73	92.80	0.20
Cuba	27,898	5.74	89.63	1.23
El Salvador	26,692	8.32	87.63	0.37
Jamaica	22,201	90.60	1.24	2.22
Peru	18,354	12.49	76.44	5.36
Paraguay	16,230	10.02	81.84	2.21
Guatemala	15,887	9.42	84.21	0.57
Uruguay	14,892	6.13	83.11	3.24
Nicaragua	14,494	6.80	86.44	0.85
Costa Rica	14,358	14.88	79.89	0.73
Panama	10,483	19.27	76.22	0.34
Bolivia	6,174	11.71	82.22	1.00
Trinidad & Tobago	5,272	93.27	2.37	0.13
Haiti	3,835	50.38	1.75	0.23
Martinique	3,238	24.52	0.80	0.19
Guadeloupe	2,508	27.31	0.68	0.16
Bahamas	2,215	71.38	22.17	0.59
Barbados	2,147	87.61	4.15	2.10
Suriname	1,404	72.29	2.35	0.21
Grenada	1,111	93.61	2.70	0.00
US Virgin Islands	1,038	95.28	0.19	0.00
Aruba	987	31.81	25.63	1.11
Guyana	824	94.30	1.09	0.12
St. Lucia	776	86.47	0.64	0.90
Cayman Islands	749	91.99	0.93	0.27
Belize	652	82.36	12.58	0.31
Antigua & Barbuda	484	85.33	1.65	4.75
Dominica	457	94.31	1.31	0.22
Turks & Caicos Islands	183	93.99	1.09	0.00
British Virgin Islands	181	84.53	2.76	0.00
Saint Vincent & Grenadines	82	93.90	1.22	0.00

#### Language

We use langid.py [44] to infer the language of each tweet, which is estimated to have an accuracy of 94% on tweets in European languages, the target of our study. The three most common languages are English (6.9 million tweets), Spanish (3.7 million), and Portuguese (1.8 million). Within the Americas, 3.7 million (out of 3.9 million) tweets are in one of these three languages. Our topic analysis focuses on these languages, which represent the most common languages spoken in North and South America. Table 1 shows the percentage of tweets in each location that are in each of these languages.

No location in the dataset is linguistically homogeneous; all have a mix of languages, and all contain at least some English. A few locations predominately contain other languages; for example, French-speaking Martinique only has about 25% coverage of tweets with these languages.

### Topic modeling

We use probabilistic topic models to extract topics or themes from the tweets. Topic models like Latent Dirichlet Allocation (LDA) [33] are considered to be user-friendly and interpretable [30], and as we discuss in this section, they can be adapted for use across multiple languages—a property that many alternative text mining methods do not have.

When a dataset contains more than one language, it is more complicated to apply LDA because different languages will be separated into different topics due to the lack of overlap in words. For some studies, like our work here, it is useful to have a model where each topic corresponds to the same concept in every language. Thus, various *multilingual* extensions to LDA have been proposed [45–47], of which the most widely used extension is the polylingual topic model [7]. This model applies to corpora containing either direct translations of documents or closely comparable documents (e.g., language-specific versions of Wikipedia articles). Analogous to LDA, each document *d* in language *ℓ* has a distribution over topics, $\theta_{d}^{\left(\ell \right)}$, which is shared across all language-specific versions of that document: $\theta_{d}^{\left(\ell \right)}=\theta_{d}^{\left(\ell^{\prime }\right)},\forall \ell^{\prime }\neq \ell$. Each topic *k* has a distribution over words specific to each language *ℓ*, $\phi_{k}^{\left(\ell \right)}$.

In this work, we apply the polylingual topic model to the English (en), Spanish (es), and Portuguese (pt) tweets in the Zika corpus. We infer topics for the entire dataset of 15 million tweets, even though we only analyze the subset of 3.9 million geolocated tweets in this work, so that topic metadata is available for all tweets in these languages in our shared dataset.

#### Synthesizing parallel data

Unlike standard data sources like Wikipedia, the Zika tweet dataset does not contain documents that are aligned across languages, so polylingual topic modeling cannot be applied to the raw data directly. To address this problem, we create translated versions of tweets using machine translation (MT) [48]. This approach was successfully used in previous topic modeling work [49].

We use the Microsoft Translator Text API to translate tweets, a service that interfaces with a proprietary neural machine translation system [50]. Translating the full corpus (priced at $10 USD per 1,000,000 characters) was beyond our budget. We translate a random sample of tweets, where each tweet from this sample is translated from its original language into the other two languages, so that each tweet has a version in all three languages. The service translated 85,214 en tweets into es and pt, 38,540 es tweets into en and pt, and 38,857 pt tweets into en and es (162,611 tweets total). During this process, the numbers of tweets was sampled such that they were proportional to the distribution of these three languages in the data, up to the number of tweets possible under our budget cap.

The polylingual topic model does not require every document to have a version in every language; the untranslated tweets can be included as well, but without alignment to other languages. In other words, the subset of 162,611 tweets that have aligned translations will help the model learn alignments in topics across languages, but the model can still be used to infer topics on the entire set of 3.9 million tweets.

An alternative approach would be to translate all tweets into a single language, such as English, and simply apply a monolingual topic model. However, this approach would completely replace the original tweets in other languages with their artificial translations, which would introduce a bias in the quality of topics across languages and may lead to language-specific trends. In contrast, the proposed approach retains all original tweets, while only using synthetic data for the purpose of learning alignments across topics.

#### Training and evaluation

We use the polylingual topic model implementation in MALLET [51] to generate topics from our synthesized parallel dataset. Tweets are preprocessed to remove stop words in each language, user names, hashtags, and URLs. We use the stop word list built into MALLET for en, and use the stop word lists for es and pt from [52]. Additionally, the words “zika” and “virus” (with its es and pt translations) are also removed, as they appeared in most tweets. All strings were made lowercase, but we did not apply stemming or de-duplication [53, 54]. After preprocessing, the corpus has an average of 13.5 tokens per tweet in en and pt and 13.7 tokens per tweet in es (30,981,491 total tokens). The number of unique words is 213,382 in en, 180,861 in es, and 198,485 in pt.

Models are trained with 2,000 Gibbs sampling iterations (a common setting [55]), with automatic hyperparameter optimization every 10 iterations. The hyperparameters were initialized to the MALLET default values (*α* = 1/*K*, where *K* is the number of topics, and *β* = 0.01, corresponding to the Dirichlet priors for the topic distributions *θ* and word distributions *φ*, respectively). We avoid extensive hyperparameter tuning by allowing the software to optimize their values automatically [56]. Automatic hyperparameter optimization is activated by adding the flags, --optimize-interval with value 10 and --optimize-burn-in with value 20 [57].

To help perform model selection, we calculate two automated evaluation metrics:

- **Coherence:** A common way to evaluate the quality of a topic is to assess whether the words in that topic are in fact related to each other. This is calculated using statistics of word co-occurrences [58]. We use normalized pointwise mutual information **(NPMI)** as the co-occurrence statistic [59], using the implementation of Lau [60]. Each topic’s coherence is measured as the average NPMI of all pairs of words in the topic. While a standard approach is to calculate the co-occurrence statistics on a large external corpus like Wikipedia, we found this to result in near-zero NPMI values when applied to the Zika topics, perhaps due to the style mismatch of Twitter or the domain specificity of Zika. We instead calculated the co-occurrence statistics from the documents being modeled [61], using the Zika tweets (excluding the automatically translated tweets) to calculate NPMI.
- **Consistency:** In a multilingual topic model, it is also important that the language-specific versions of a specific topic *k* are all related to the same concept. We quantify this using matching translation accuracy **(MTA)** [45], which measures the percentage of words in one language’s version of a topic *k* that are a direct translation of a word in another language’s version of the same topic. To check for direct translations, we use the dictionary from Rolston and Kirchoff [62] and also count identical strings as translations. The dictionary does not contain direct translations between es and pt, so we only calculate this metric for the language pairs (en, es) and (en, pt).

Both metrics define each topic as the set of its *M* most probable words. In our experiments, we used a cardinality of *M* = 10, a commonly used value [63].

To better understand how well our machine translation approach learns consistent topics, we conduct the MTA evaluation using varying amounts of available translation pairs. To do this, we randomly sample a subset of translation pairs to make available during training. The purpose of this experiment is to characterize how many translations are needed to achieve good performance.

#### Model selection

We train the topic models with varying numbers of topics *K* as 10, 25, 50, 75, and 100. We experiment with three conditions for constructing training data:

- **MT (all tweets):** We apply the polylingual topic model on the full set of 3.9 million tweets, where 162,611 tweets are aligned with translations into the other two languages, while the remaining 3.8 million tweets do not have language alignments.
- **MT (translations only):** We train the topic model on only the subset of 162,611 translated tweets, as an alternative to training on all tweets. This is done to test whether having a mix of translated and untranslated tweets in the corpus affects the consistency of topics across languages.
- **Word replacement:** Because machine translation is expensive, researchers have considered “cheap” translation alternatives based on substituting words on a word-by-word basis without inferring correct translations based on context [64]. This method was determined to be a suitable alternative to full MT in Lucas et. al. [49].

For the word replacement approach, we synthesize tweets by substituting each tweet’s words with the corresponding words in the bilingual dictionaries, using a simple heuristic to select the best word when multiple translations are available, as simple heuristics have been showed to be sufficient in similar work [65]. Specifically, for each word token, if there are multiple translations available, we randomly sample which translation is substituted.

To choose the best topic model, we use a combination of qualitative and quantitative (NPMI and MTA) approaches, motivated by our requirements that the topics must be identifiable as concrete concepts that we can reference in the downstream analysis. Some quantitative metrics like log-likelihood have been shown to be negatively correlated with topic quality as perceived by humans [66], so we focus on NPMI as a metric designed to capture human interpretability [59] and MTA as a way to make sure the topics are aligned across languages. MTA has limitations discussed in [63], and even NPMI has only a moderate correlation with human judgments [67], so rather than fully relying on these metrics to choose the best model, we use these metrics as a guide to understand the general effects of different hyperparameters, while also qualitatively examining the output to find a model that yields interpretable topics that can be identified as specific concepts. This involves reading through each topic’s list of top words and identifying whether a common theme exists among the grouping of words, and seeing the degree to which individual words in the topic belong with the group (that is, are coherent). Qualitative judgments of topic quality can be quantified, for example on a Likert scale [66], but we did not quantify topic quality in this study. Thus, the final model we select may not be the single best model under particular quantitative measurements, but we observe that the topic models typically identify the same set of themes and vary mainly in the amount of redundancy across topics and coherence within individual topics, and thus selection of a different model should not greatly affect the general findings.

### Spatiotemporal analysis

To better understand and contextualize the results from our topic model, we additionally examine spatiotemporal trends: *when* and *where* are people tweeting about Zika?

#### Overall tweet volume

We measure the volume of tweets in different locations and time intervals. For each location, we divide the number of Zika tweets in that location by the number of tweets from that location in the control sample of 42 million tweets. This adjusts the counts for the overall activity of Twitter in that location, and can be interpreted as being proportional to a *per-capita* estimate of tweets in that location that mention Zika [13]. *Adjusted volume* refers to this measurement.

#### Comparison to incidence rates

Additionally, we measure the association between Zika-related tweet volume in each location with actual Zika incidence in each location. While one might expect more Zika-related social media activity in areas with high Zika incidence, there can be geographic disparities in health knowledge that might affect this hypothesis [68], and prior work on influenza in Twitter has found that tweet volumes are driven more by news media than by actual disease incidence [19]. Research on risk perception has found that while actual disease risk may be a factor in risk perception, news media attention is also a factor [69]. Thus, in terms of studying risk communication, it is of interest to understand the degree to which online social media discourse, and various topics within that discourse, are related to actual levels of Zika in each population.

To measure the association between disease incidence and tweet activity for Zika, we compare the Zika tweet volumes to cumulative Zika case counts in each location, obtained from the Pan American Health Organization (PAHO, part of WHO) for the final week represented in our dataset (October 27, 2016) [70]. We measure the association using Spearman’s rank correlation (*ρ*).

In addition to comparing overall tweet volumes to incidence rates, we correlate topic-specific volumes to incidence rates to understand which topics are associated with Zika incidence. We follow the same procedure for calculating the correlations, but the adjusted volume in each location is multiplied by the average topic probability for that location (described in the next section).

#### Topic prevalence

We examine temporal and geographic trends of the specific topics identified by the polylingual topic model.

Within each location, we calculate the average proportion of each topic in that location. For a location *l* and topic *k*, the average topic probability is: $\frac{1}{|D_{l}|}\;\;\sum_{d\in D_{l}}\;\;\theta_{dk}$, where *D*_*l*_ is the set of tweets from location *l*.

To calculate topic prevalence over time, we calculate adjusted topic proportions for each week. The adjusted topic proportion for a topic *k* in week *w* is: $\sum_{l}\sum_{d\in D_{lw}}\frac{\theta_{dk}}{N_{l}}$, where *D*_*lw*_ is the set of tweets posted in week *w* from each location *l*, and *N*_*l*_ is the number of tweets from location *l* in the control sample.

## Results

### Topic modeling

#### Model selection

Table 2 shows the evaluation metrics across the different models. For clarity, the table only shows the results averaged across the three languages (for NPMI) and two language pairs (for MTA), but there are some differences between languages. English usually has the highest NPMI, possibly because it is the most prevalent language in the corpus. Using all tweets instead of only translated tweets improves NPMI for English and Portuguese, but hurts Spanish, so on average the two versions of the dataset have similar mean NPMI. Among language pairs, (en, es) have consistently higher MTA than (en, pt).

**Table 2:** Topic model evaluation (NPMI and MTA) for different training conditions and different numbers of topics (*K*). The two “MT” settings use a full machine translation system, while the “word replacement” approach approximates machine translation by simply replacing the words with entries in a bilingual dictionary.

*Training data*	*K*	*NPMI*			*MTA*		
		Mean	SD	Max	Mean	SD	Max
MT (all tweets)	10	.185	.063	.291	3.15	1.85	6.00
MT (translations only)	10	.101	.058	.202	7.45	1.72	9.00
Word replacement	10	.130	.075	.289	3.30	2.07	6.50
MT (all tweets)	25	.112	.082	.295	3.30	2.44	7.50
MT (translations only)	25	.111	.062	.247	7.44	1.41	9.50
Word replacement	25	.126	.086	.327	3.28	2.13	7.50
MT (all tweets)	50	.096	.097	.342	3.22	2.05	8.50
MT (translations only)	50	.098	.070	.306	7.37	1.42	10.00
Word replacement	50	.126	.085	.361	3.39	2.12	8.50
MT (all tweets)	75	.150	.086	.424	2.77	1.89	9.00
MT (translations only)	75	.089	.076	.346	7.12	1.51	10.00
Word replacement	75	.124	.085	.363	3.61	2.08	9.00
MT (all tweets)	100	.127	.082	.404	2.26	1.58	7.00
MT (translations only)	100	.078	.068	.327	6.58	1.47	10.00
Word replacement	100	.113	.086	.384	3.49	1.94	9.00

Comparing the MT model learned from all tweets compared to the model learning from only the translated tweets, we do see an expected drop in language consistency (MTA), but an increase in coherence (NPMI). Interpreting MTA is somewhat difficult because two words across languages may be related even if they are not direct translations, and we would not expect MTA to always be high, as some topics will naturally vary across languages due to the ways they are discussed in different countries. Instead, we want topics to have at least some direct association across languages, and we qualitatively confirm that they do appear consistent across languages. To put the MTA scores into context, we calculated MTA across different pairs topics (e.g., topic *k* in en and topic *j* ≠ *k* in es) to measure how often topics would contain the same words by coincidence. The average was 1.12, about 3 times less than the MTA of 3.22 when using all tweets with *K* = 50, thus even models with relatively low MTA are still capturing alignments across languages.

Comparing the MT models to the word replacement model, we observe that the MTA scores for the word replacement model are more similar to the MT model trained on the full corpus (which mostly contains untranslated tweets) rather than the MT model trained only translated tweets. The MTA scores are slightly higher with word replacement than with MT on all tweets, which could be due to having more translations with the word replacement method, though we caution that MTA scores might be inflated for the word replacement method since the evaluation metric is based on the same resource used for training the model in the first place. The NPMI results vary by topic; sometimes word replacement has the highest NPMI and other times MT with all tweets gives the highest NPMI. Qualitatively, we find that the word replacement model learns similar topics to using full MT, but we do observe artifacts caused by “cheap” translation that are not present when using full MT. For example, the word “computer” is a top word in many topics using word replacement but not using MT, which seems to be caused by the dictionary-based translation using the computer sense of the word “viruses” rather than the biology sense. (While “virus” was in our stop word list, its pluralization was not).

Comparing the different settings of *K*, we first note that our downstream analysis does not cover all *K* topics, because we only analyze the subset of topics that are interpretable and identifiable as coherent concepts. It is common for many topics to be incoherent or noisy, especially when modeling short texts like Twitter [71], and only a subset are used for further analysis [34]. Therefore, the average topic quality does not matter as much as the number of high-quality topics. Even though larger *K* results in lower average coherence due to more topics with high noise, the distribution of NPMI at the top is better than at *K* = 10 or *K* = 25, with a much higher maximum NPMI when *K* is large. We also observe that there are more fine-grained topics when *K* is larger, which is helpful for analysis. When *K* is larger than 50, we start to see duplicated topics, which creates challenges for downstream analysis because the same concept can be represented across multiple topic labels. Additionally, MTA tends to drop once *K* is larger than 50.

After comparing models, we select the MT model trained with all 3.9 million tweets using *K* = 50, due to its balance of high coherence, acceptable cross-lingual consistency, and reduced risk of translation error.

#### Sensitivity to translation availability

**Fig 1:**
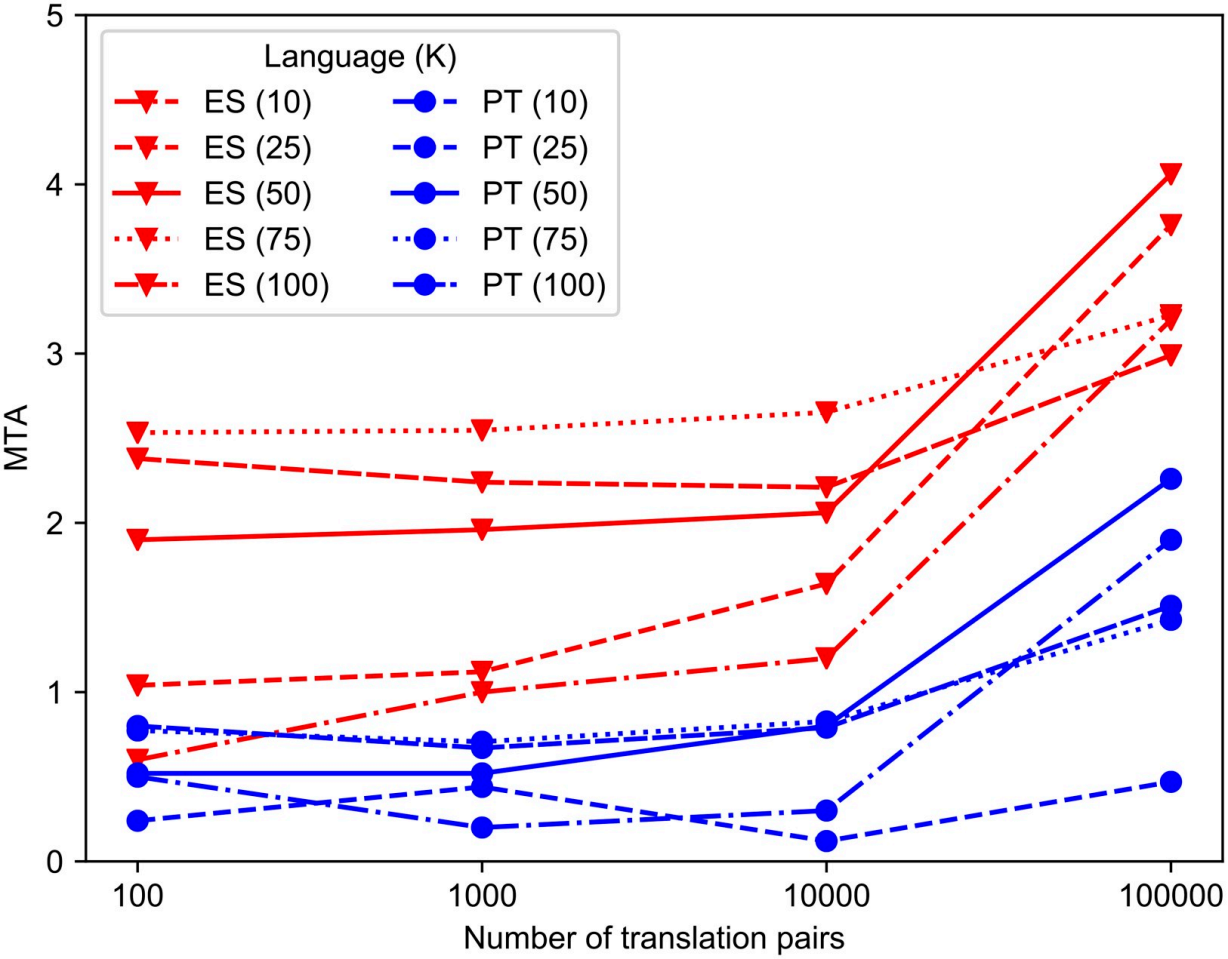
Crosslingual consistency versus number of translation pairs. Learning curves showing how crosslingual consistency of topics, measured by MTA, varies with the number of pairs of translated documents in the corpus.

Fig 1 shows learning curves on a log_10_ scale when different numbers of MT translation pairs are available. The curves show that crosslingual consistency, measured by MTA, is substantially higher when using all 100,000 translation pairs compared to the next-highest amount of 10,000 pairs. It is therefore not clear from these results if acceptable performance could be achieved with fewer translations.

#### Topic discovery

Among the 50 topics, we focus on a subset of topics that were relevant to Zika and qualitatively meaningful to the research team. Many topics contain generic terms about general news and are not identifiable as a particular theme. Some topics are coherent but excluded from the analysis because they are not related to Zika, such as topics about other mosquito-borne diseases like dengue.

Two researchers (MJP and ARD) independently examined and labeled the 50 topics. The annotators then compared labels and discussed the topics, deciding on a final set of labeled topics. Table 3 shows the 14 topics that were identified for this study. The topics show that people tweet about Zika research, including vaccination research and links to other disorders, microcephaly and Guillain-Barré syndrome, travel advisories and protection against mosquitos, and discourse around the Olympics and political response, among other topics.

**Table 3:** The top words representing 14 topics (labeled manually) aligned across three languages. The numbers in parentheses after each language indicate the overall topic proportion (i.e., the average value of *θ*_*k*_ across documents in that language, where a higher value means it appears more in the corpus). The rank correlation (*ρ*) with per-country incidence rates is shown after each topic number.

**Conspiracy** **Theories** Topic 1 (*ρ* = .154)	en (.016) es (.023) pt (.011)	bill conspiracy gates foundation rockefeller fear media theories people vaccine mosquitoes hoax rockefeller casos vía dengue mosquito alerta patentado embarazadas monsanto fundación país pra dengue só gente tá brasil microcefalia mundo vou rockefeller nao nois sai to conspiração
**Environmental** **Concerns** Topic 4 (*ρ* = .145)	en (.045) es (.023) pt (.012)	mosquitoes spraying genetically fight millions modified bees south florida mosquitos spray mosquitos combatir casos millones brasil mosquito genéticamente vía modificados transgénicos mosquitos mosquito dengue combater só brasil milhões pra cara q tá chikungunya
**Negative** **Effects** Topic 10 (*ρ* = .213)	en (.026) es (.056) pt (.068)	microcephaly cases linked link syndrome study guillain disorder barre evidence colombia guillain barré casos síndrome microcefalia oms barre relación colombia estudio confirma casos microcefalia síndrome suspeitos guillain barré colômbia confirma saúde registra doença
**Viral Testing** Topic 12 es (.030) (*ρ* = .055)	en (.034) pt (.050)	blood test fda testing urine donations emergency saliva florida supply screening donated areas sangre prueba brasil orina saliva salud dengue pruebas detectar casos vía donaciones teste saúde sangue saliva fiocruz planos testes rápido urina dengue diagnóstico exames brasil
**Mosquito** **Advisories** Topic 21 (*ρ* = .178)	en (.015) es (.014) pt (.007)	mosquito repellent bug spray insect protect prevent bites mosquitoes repellents news deet mosquito repelente casos mosquitos vía repelentes embarazadas prevenir dengue by evitar auto mosquito q repelente pra to tá ta dengue vc gente vou brasil deus pq proteger repelentes mlk
**Olympics** Topic 25 (*ρ* = −.066)	en (.051) es (.052) pt (.041)	rio olympics fears olympic games concerns due mcilroy rory athletes day world golfer janeiro juegos olímpicos río rio janeiro brasil atletas temor oms miedo riesgo olímpico vía mundo rio jogos olimpíadas olímpicos atletas medo olimpíada brasil causa janeiro olímpico mundo
**Research** Topic 28 (*ρ* = .19)	en (.036) es (.040) pt (.055)	brain study infection cells fetal damage microcephaly babies scientists researchers brains adult estudio científicos feto cerebral cerebro células infección casos investigadores descubren causar estudo cientistas microcefalia pesquisa cérebro bebês pesquisadores infecção brasil células
**Vaccination** Topic 32 (*ρ* = .135)	en (.037) es (.033) pt (.026)	vaccine trials human vaccines scientists develop race testing news development biotech research vacuna humanos vacunas dengue pruebas año brasil vía prueba ensayos salud ee oms casos vacina mim pra q brasil mina eua mó humanos vacinas testes fita tava deu instituto cientistas
**US Politics** Topic 34 (*ρ* = .013)	en (.070) es (.020) pt (.017)	bill funding senate gop house congress democrats planned parenthood pass republicans dems congreso obama combatir millones fondos casa pide lucha dólares casos senado financiación combater congresso us milhões financiamento casa obama eua combate governo pede dinheiro
**Reproductive** **Health** Topic 38 (*ρ* = .088)	en (.029) es (.032) pt (.028)	abortion women pope contraception latin america birth crisis countries access rights control aborto mujeres países onu anticonceptivos papa américa afectados acceso pide vía casos latina aborto mulheres grávidas onu países américa epidemia papa q brasil defende latina pra
**Microcephaly** Topic 39 (*ρ* = .146)	en (.055) es (.036) pt (.015)	birth defects born baby microcephaly linked babies defect related link brazil brain cdc severe microcefalia bebé bebés nace nacimiento defectos mujer españa recién luz madre causa nacido microcefalia bebês bebê brasil pra mãe causa só defeitos nascimento q ta to confirma eua
**Florida** **Outbreak** Topic 41 (*ρ* = .189)	en (.034) es (.013) pt (.008)	miami florida beach cases scott gov local county dade rick travel officials zone area governor miami florida casos mosquitos beach salud unidos transmisión gobernador cdc zona casos miami saúde flórida eua mosquitos transmissão caso estados mosquito florida
**Advisories** Topic 45 (*ρ* = .355)	en (.029) es (.026) pt (.036)	pregnant women travel areas cdc avoid affected countries sex pregnancy airlines latin hit mujeres embarazadas zonas sexo recomienda países oms cdc evitar viajar seguro unidos viajes mulheres grávidas eua oms países américa evitar saúde sexo cdc áreas gravidez latina brasil
**Emergency** **Declaration** Topic 48 (*ρ* = .203)	en (.037) es (.067) pt (.042)	health emergency world public global organization declares international declared spread emergencia oms salud mundial declara sanitaria internacional organización global pública saúde oms emergência mundial organização global pública declara alerta brasil internacional

Of the 36 topics that were not included for analysis, most contain a mix of common words that seem to be associated with general news rather than a specific identifiable theme. Examples of such topics include, “pregnant health women olympics cases cdc news brazil mosquito florida,” “health news today brazil pregnant cases spread women morning break,” and “fight google spread news control brazil map outbreak working million.”

Some topics were coherent in one language but not consistent across languages. For example, the English-language topic, “dr today pm questions live join director disease infectious cdc,” appears to represent “group chats” on Twitter [72], but the words in the other languages were more general and did not appear to be specific to chats.

Finally, a small number of topics were coherent but not relevant to Zika, such as this topic containing several different infectious diseases, “ebola flu aids people disease west hiv swine world cancer dengue diseases nile.”

### Analysis of tweet volume

**Fig 2:**
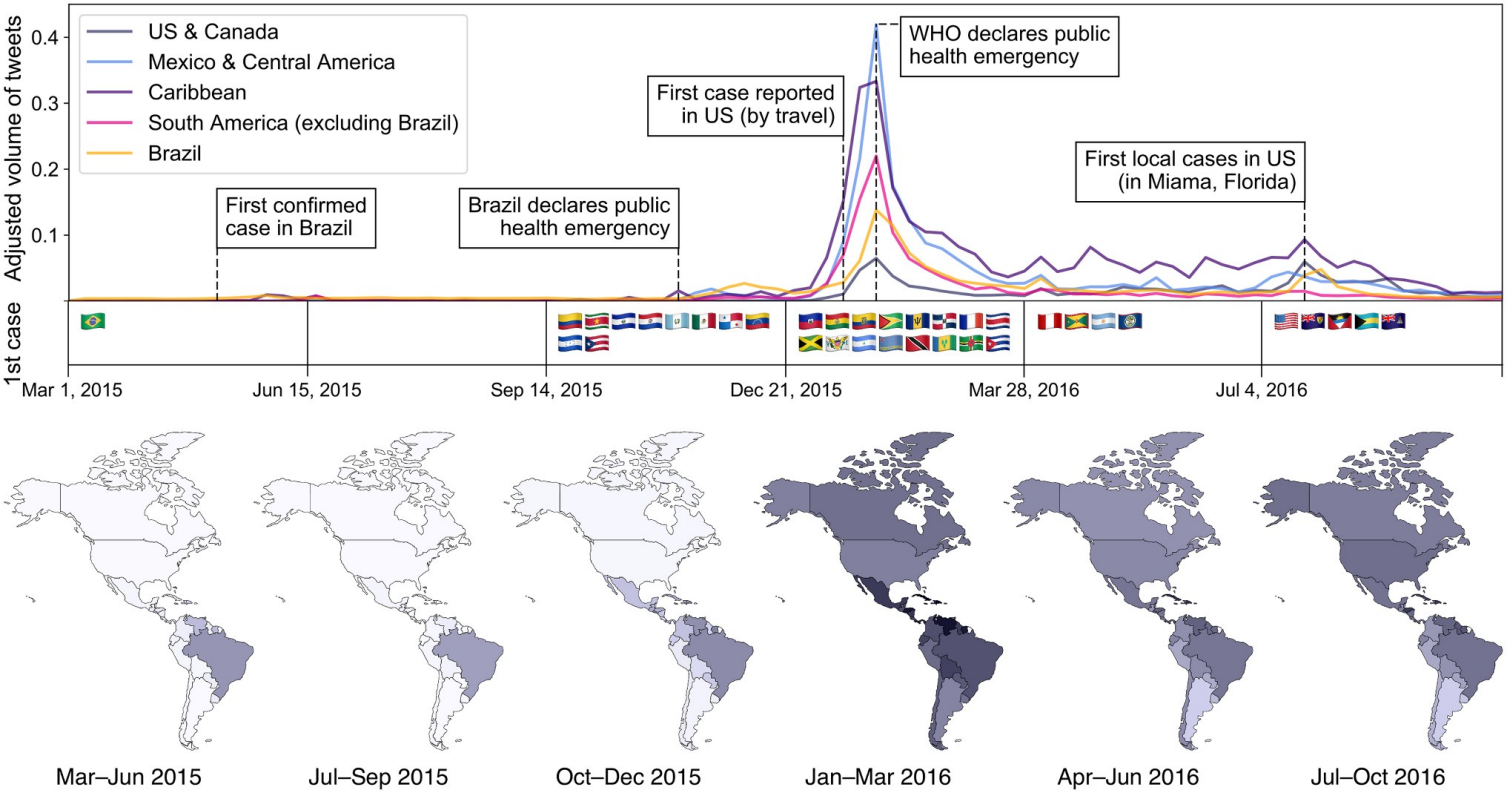
Volume of Zika tweets across time and place. *Top:* The adjusted volume of Zika-related tweets in five geographic regions per week. The country flags below the line plots indicate the time window in which each country reported its first Zika case. *Bottom:* Tweet volume in each country during six time windows spanning our data collection. Darker shading indicates higher volume; the color scale is on a log_2_ scale.

Fig 2 shows the adjusted volume of Zika tweets from different locations, mapped for individual locations during broad time intervals, as well as for broad geographic regions during weekly intervals.

There are few Zika tweets throughout most of 2015. Twitter activity rises in November and December, peaking in January and February. While the bulk of tweets are from the US, after adjusting for total volume, there is actually relatively little Zika discussion in the US. Instead, tweets are more likely to come from Brazil in 2015 and from Central America and the Caribbean in 2016.

#### Comparison to world events

The bottom of Fig 2 provides a summary of key events in this Zika outbreak, including when each country first reported a locally-transmitted case of Zika (i.e., not transmitted while traveling in another country). The timeline is shown on the same x-axis for direct comparison to Zika-related Twitter activity.

The first confirmed case was reported in Brazil on April 29, 2015, which is followed by a small and temporary rise in tweet activity in Brazil and its neighbors. Zika is not reported outside of Brazil until October 16, when it was confirmed in Colombia. Tweet activity remained low during this period.

The next observable rise in activity occurs the week after Brazil’s Ministry of Health declared a national public health emergency, on November 11. The rise is largest and most sustained in Brazil, but there is also a rise in activity elsewhere in South and Central America.

Tweet activity in the US and Canada continued to stay low until the first case was reported in the US, on January 17, when a Zika-infected baby was born in Hawaii, whose mother had recently lived in Brazil. The baby was born with microcephaly and the news was widely reported. Tweet volumes rose this week in every location.

The largest peak in every location corresponds to the week that the World Health Organization (WHO) declared the Zika outbreak to be an international public health emergency, on February 1. Volumes gradually decline over the weeks following this peak, though they remain substantially higher than in 2015.

There is a second rise in tweet activity in 2016 following the announcement of the first locally-transmitted cases in the US, where four cases were confirmed in Miami, Florida on July 29. This rise in tweets was most pronounced in the US, where the number of tweets that week (169,257 tweets) is nearly as high as during the February peak (177,651 tweets). US volumes remained elevated for six weeks after this news.

#### Relation to incidence rates

The high relative tweet volume in Central America and the Caribbean and low volume in countries like the US and Argentina seems to correlate with the rate of Zika incidence in those areas, as we now describe. According to data from PAHO [70], the incidence rate was 0.04 cases per 100,000 people in the US, 4.05 in Mexico, 119.08 in Central America, 318.02 in the Latin Caribbean, 241.43 in the non-Latin Caribbean, 147.95 in Brazil, 122.37 in northwestern South America, and 3.48 in southern South America.

Comparing incidence rates in individual countries to adjusted Twitter volumes, we find a moderate rank correlation of *ρ* = .398 (*p* = .009). Thus, people in areas more affected by Zika tend to tweet more about it.

### Analysis of topic prevalence

We examine temporal and geographic trends of the specific topics identified by the topic model, focusing on the 14 topics in Table 3.

#### Geographic trends

**Fig 3:**
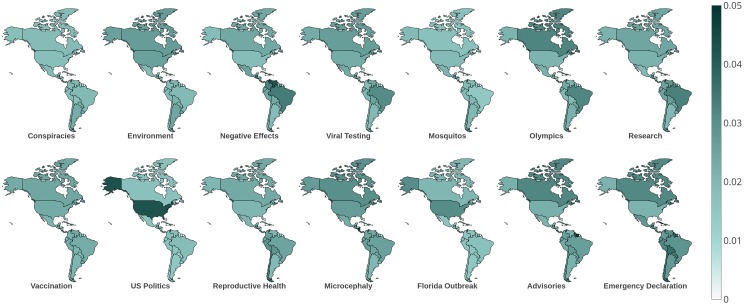
Topic prevalence by location. Geospatial distribution of the topics from Table 3. Darker shading indicates a higher average probability of the topic in that location.

Fig 3 shows the average topic probabilities in each location, while Table 4 summarizes the results by displaying the three highest-probability topics in each location. We observe a few patterns. Certain topics, especially *Vaccination* and *Microcephaly*, are discussed everywhere and are not associated with particular locations. Unsurprisingly, the US-centric topics *US Politics* and *Florida Outbreak* are more common in the US than elsewhere. The *Olympics* topic is most common in Brazil (where the Olympics were held) and Canada. Finally, the *Negative Effects* topic, which includes Guillain-Barré syndrome, is most common in the countries with higher incidence of these syndromes like Colombia.

**Table 4:** The top three topics with the highest average topic probabilities in each location.

Country	Top Three Topics
Antigua and Barbuda	Microcephaly, Advisories, Environment
Argentina	Conspiracies, Environment, Olympics
Aruba	Emergency Declaration, Negative Effects, Microcephaly
Barbados	Conspiracies, Advisories, Vaccination
Belize	Florida Outbreak, Microcephaly, Advisories
Bolivia	Reproductive Health, Viral Testing, Emergency Declaration
Brazil	Viral Testing, Olympics, Research
British Virgin Islands	Florida Outbreak, Conspiracies, Environment
Canada	Olympics, Environment, Viral Testing
Cayman Islands	Environment, Florida Outbreak, Viral Testing
Chile	Reproductive Health, Mosquitos, Conspiracies
Colombia	Negative Effects, Reproductive Health, Olympics
Costa Rica	Reproductive Health, Olympics, Research
Cuba	Emergency Declaration, Vaccination, Olympics
Dominica	Emergency Declaration, Research, Negative Effects
Dominican Republic	Olympics, US Politics, Florida Outbreak
Ecuador	Olympics, Vaccination, Research
El Salvador	Olympics, Reproductive Health, Negative Effects
Grenada	Reproductive Health, Negative Effects, Advisories
Guadeloupe	Research, Advisories, Negative Effects
Guatemala	Negative Effects, Reproductive Health, Research
Guyana	Emergency Declaration, Advisories, Research
Haiti	Florida Outbreak, Microcephaly, Reproductive Health
Honduras	Mosquitos, US Politics, Environment
Jamaica	Negative Effects, Mosquitos, Microcephaly
Martinique	Advisories, Emergency Declaration, Florida Outbreak
Mexico	Viral Testing, Vaccination, Conspiracies
Nicaragua	Microcephaly, Vaccination, Viral Testing
Panama	Microcephaly, Vaccination, Negative Effects
Paraguay	Conspiracies, Olympics, Emergency Declaration
Peru	Reproductive Health, Vaccination, Research
Puerto Rico	US Politics, Environment, Florida Outbreak
Saint Vincent and the Grenadines	Mosquitos, Florida Outbreak, Reproductive Health
St. Lucia	Conspiracies, Mosquitos, Viral Testing
Suriname	Advisories, Research, Emergency Declaration
The Bahamas	Florida Outbreak, Environment, Mosquitos
Trinidad and Tobago	Emergency Declaration, Microcephaly, Mosquitos
Turks and Caicos Islands	Mosquitos, Advisories, Florida Outbreak
US Virgin Islands	Viral Testing, Advisories, Florida Outbreak
United States	US Politics, Florida Outbreak, Environment
Uruguay	Conspiracies, Vaccination, Olympics
Venezuela	Negative Effects, Vaccination, Research

Comparing topic-specific volumes to country-specific Zika incidence rates from PAHO, we find that topics vary in the degree to which they are correlated with Zika incidence (Table 3). The topic with the highest correlation with Zika incidence is *Advisories* (*ρ* = .355), followed by *Negative Effects* (*ρ* = .213) and *Emergency Declaration* (*ρ* = .203). The topics with negative or near-zero correlations are *Olympics* (*ρ* = −.066) and *US Politics* (*ρ* = .013), which are arguably less directly related to the disease than most of the topics with higher correlations.

Some topics have relatively low correlation with Zika incidence and may instead be influenced by other factors. For example, the *Reproductive Health* topic may attract more discussion and debate in countries with higher levels of Catholicism. The country with the highest volume of this topic is Chile, which has a high Catholic population [73]. Although other nearby countries also have high or higher levels of Catholicism, Chile differs in having very low Zika incidence according to the PAHO data.

#### Temporal trends

**Fig 4:**
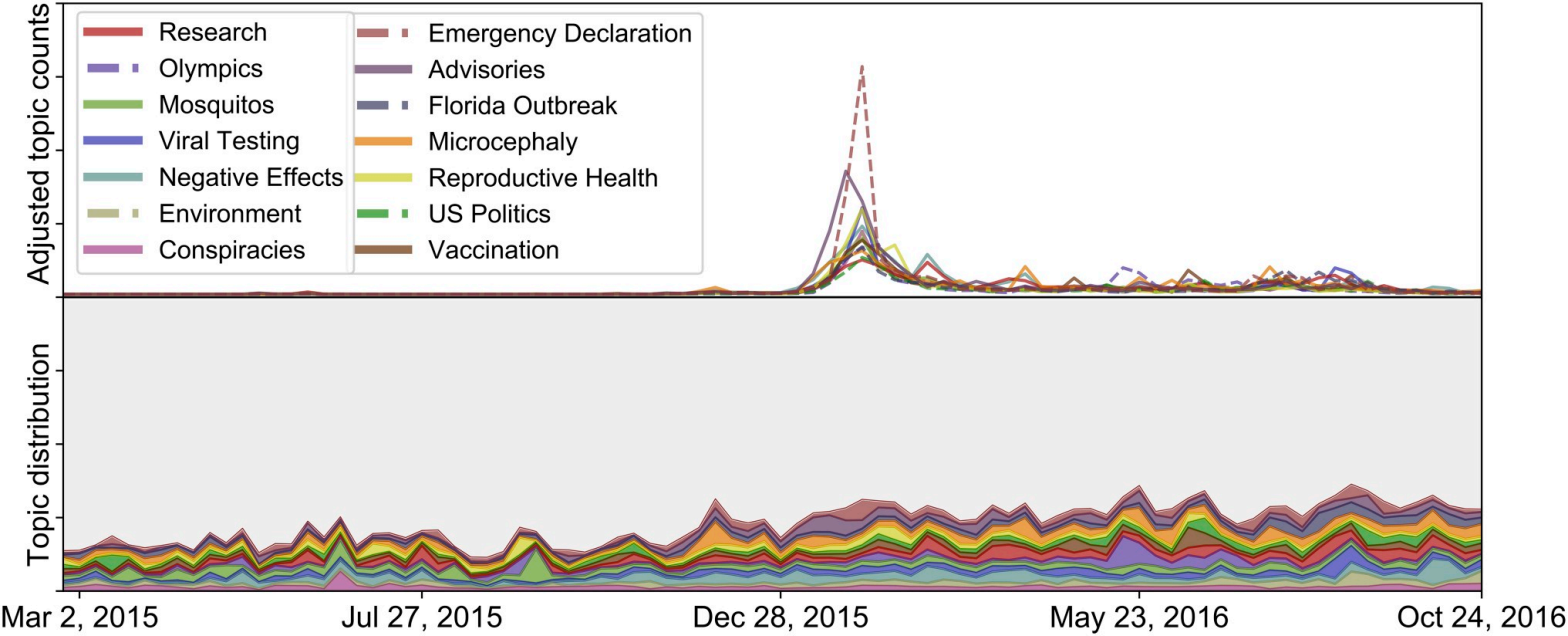
Topic prevalence by time. The volume and distribution of the topics from Table 3 per week. The adjusted counts of each topic are shown on top; dashed lines are added for readability and do not have a special meaning. The adjusted counts are normalized to sum to 1 on the bottom; the shaded gray area represents the proportion of the 36 other topics outside of the 14 topics in Table 3.

Fig 4 shows the volume and distribution of topics over time. The top half of the figure shows the adjusted topic proportions directly, while the bottom renormalizes the adjusted proportions to sum to 1 so that the distribution over the topics can be seen. Normalization allows for the observation of topic proportions in 2015 when the overall volumes are very low.

As noted in the overall volume analysis, the peak Twitter volume occurs in the week the WHO declared a public health emergency; as would be expected, the *Emergency Declarations* topic is the driver of this peak. Interestingly, the *Advisories* topic rises in the weeks preceding this peak.

Smaller spikes from various topics occur in tandem with specific events. A spike in the *Reproductive Health* topic occurs in February when Pope Francis declared that contraception (typically disallowed in Catholicism) is acceptable if used to protect against Zika [74]. A spike in both the *Negative Effects* and *Research* topics corresponds to the publication of a *New England Journal of Medicine* study providing new insight into the link between Zika and microcephaly [75]. A rise in the *Olympics* topic in May 2016 coincides with a letter that a group of 150 physicians sent to the WHO about the risks of holding the Olympics in Brazil [76].

## Discussion

The goal of this study is to introduce and characterize our comprehensive Twitter dataset, which contains all tweets mentioning Zika over a 20-month period. Our exploratory analyses illustrate what, where, and when Zika-related information is posted on Twitter. It is clear that Zika is widely discussed, and discussed differently around the world. Zika discourse in each location appears to be associated in part with the incidence of Zika in that location, with certain topics being discussed more or less in areas with high versus low levels of Zika. These variations could have implications for what topics of online discussion would be informative for disease modeling [25].

Since there is not currently an effective vaccine or medication to combat the Zika virus, information campaigns to promote other protective behaviors, like the use of insect repellent and contraception, are the primary public health control measures for this outbreak. In a limited way, through the volume of tweets, our study provides insight into the levels of awareness and discussion of different Zika-related topics. An observation from our temporal analysis is that tweet activity often follows an external advisory or news event, but discussion following such events is not sustained, and tweet activity regarding a particular event drops quickly. Most of the spikes we describe in the Results section are only elevated for a single week. The *Olympics* topic was elevated for two weeks during debate over its role in Zika control before dropping back down to baseline levels; the *Florida Outbreak* topic was elevated for about three weeks during the initial news cycle, but dropped down long before the risk had dissipated. This pattern has also been observed in tweet activity regarding influenza, which tends to drop quickly after an initial spike in interest, even if infection levels remain elevated [19]. Understanding how interest in and attention to health issues can be affected by external events and media, and understanding the dynamics of public awareness, is critical for the design of public health messaging, and this dataset may help inform such efforts.

An important takeaway from this study is that social media content is geographically widespread and geographically varied, and multilingual text mining methods can help us measure this variation. While the US is the dominant source of tweets in the dataset, after calculating per-capita volumes Zika is actually discussed far less in the US than in other American locations. Moreover, the topics of discussion that are prevalent in the US are quite different from topics discussed elsewhere. Studies of Zika-related social media that focus on English-language data, as the content analyses we cited in our literature review have done, will not accurately represent the content of social media in places most affected by the disease. For example, Table 1 shows that fewer than 3.5% of tweets from Venezuela are in English, while our multilingual approach was able to cover 95% of Venezuelan tweets. The importance of multilinguality may also apply to the study of social media on other tropical diseases like dengue and chikungunya.

As a methodological contribution, we experiment with using the polylingual topic model [7] as a tool for content analysis across multiple languages. This methodology allows for the study of text in multiple languages even if the research team is not fluent in every target language, as would be required for a traditional, manual content analysis. We compare different approaches to synthesizing parallel data for learning topic representations that are crosslingually consistent. Importantly, our experiments show that while coherence depends on the amount of parallel training data, acceptable performance can still be achieved when translating only a subset of the entire corpus using machine translation, meaning that this approach can be used even under resource limitations. We also consider “cheap” translation methods based only on word substitution [49, 64], and while this approach can lead to mistranslations due to incorrect word senses, our results suggest that this could also provide acceptable performance. One possible solution that combines the advantages of the different approaches would be to use simple word translations when tweets do not contain polysemous words (i.e., words with multiple senses), and use full machine translation for tweets only when needed to resolve ambiguity.

### Limitations

Social media research is often fraught with challenges of reliability and validity [77]. While we showed patterns of information sharing in Twitter, we cannot draw conclusions about how that affects patterns of information consumption and broader awareness among Twitter users, let alone the general population. We further discuss two limitations particular to the data and methods used in this study.

First, a concern with Twitter research is the degree to which Twitter users are a representative sample of a broader population, which limits the scope of conclusions that can be drawn [77]. While the demographic coverage of Twitter is reasonably well understood for the United States [78], less is known about many of the countries covered in our dataset. Not only might demographic biases skew our results, but the biases are unknown and may be inconsistent across the population in our study. For example, while country inference with Carmen was estimated to have a 90% accuracy overall, its accuracy has not been measured on the specific set of countries used in this study. Previous work has shown that geolocation systems can be demographically biased against lower population areas [79].

Second, while various metrics exist to evaluate topic models, as we employ in this work, there is still a large amount of subjectivity involved in performing model selection, choosing the number of topics, and interpreting the topics. How to reliably ground topics in meaningful concepts remains an open challenge [80]. For measuring crosslingual consistency, topic coherence metrics like NPMI have been extended to crosslingual settings [63]. This type of approach has advantages over MTA because it captures the relatedness of words across languages, even if they are not direct translations. However, this requires a parallel corpus of aligned documents. One option is to use the synthesized documents for evaluation, but this could be an unreliable measurement because translation errors would not be identified as errors. Another option is to use an external corpus like Wikipedia to calculate NPMI. We did attempt to use Wikipedia, but due to the mismatch in the domain (Wikipedia articles on a vast range of subjects, versus tweets narrowly focused on the subject of Zika), the NPMI values were all close to zero and difficult to interpret. Therefore, we opt to use a combination of NPMI to measure monolingual coherence and MTA to measure crosslingual consistency, despite their respective weaknesses.

An additional topic modeling limitation is related to the effects of data on model training. We intentionally include retweets and duplicate tweets in the data, as we are interested in measuring the volume of each topic and if many people are tweeting the same or similar text, that is important to capture. However, topic models are known to be biased if the dataset includes duplicated text, so training them with duplicate tweets is a limitation of this approach [54].

### Conclusion

We introduce and explore a new dataset of tweets mentioning Zika, which appears to be a rich source of information about the Zika virus, with high variability in activity and content across time and place. We share (via identifiers) our collection of over 15 million tweets along with our inferred metadata and topics, with the hope that this resource can foster further research on this subject in the community, available at: https://doi.org/10.6084/m9.figshare.6025697.v1.

Prior research (e.g., [28]) has used limited Twitter data to identify important actors, concepts and locations to describe changes in spatiotemporal patterns on topics with respect to Zika. Our work shows how topics can be explored on a more extensive global scale to allow the public health community understand variations in topics across countries. These data contribute importantly to understanding of disease transmission, disease interventions, and public health communication.

Social media analyses are often limited in scope to a particular language or location. While this is often done for valid reasons, our study shows that wider comparisons may be necessary to understand the full picture. The adoption of tools capable of multilingual analysis at large scale, such as the multilingual topic modeling approach we use and extend in this project, creates important new possibilities for research.
